# The effects of Taurine supplementation on inflammatory markers and clinical outcomes in patients with traumatic brain injury: a double-blind randomized controlled trial

**DOI:** 10.1186/s12937-021-00712-6

**Published:** 2021-06-08

**Authors:** Mahsa Vahdat, Seyed Ahmad Hosseini, Farhad Soltani, Bahman Cheraghian, Masih Namjoonia

**Affiliations:** 1 Nutrition and Metabolic Disease Research Center, Ahvaz Jundishapur University of Medical Sciences, Ahvaz, Iran; 2 Department of Nutrition, School of Allied Medical Sciences, Ahvaz Jundishapur University of Medical Sciences, Ahvaz, Iran; 3 Department of Anaesthesiology and Critical Care, School of Medicine, Ahvaz Jundishapur University of Medical Sciences, Ahvaz, Iran; 4 Department of Statistics and Epidemiology, School of Public Health, Ahvaz Jundishapur University of Medical Sciences, Ahvaz, Iran

**Keywords:** Traumatic brain injury, Inflammation, Taurine, Clinical outcome, TBI

## Abstract

**Background:**

Traumatic brain injury is a public health concern and is the main cause of death among various types of trauma. The inflammatory conditions due to TBI are associated with unfavorable clinical outcomes. Taurine has been reported to have immune-modulatory effects. Thus, the aim of this study was to survey the effect of taurine supplementation in TBI patients.

**Methods:**

In this study, 32 patients with TBI were randomized into two groups. The treatment group received 30 mg/kg/day of taurine in addition to the Standard Entera Meal and the control group received Standard Entera Meal for 14 days. Prior to and following the intervention, the patients were investigated in terms of serum levels of IL-6, IL-10, hs-CRP and TNF-α as well as APACHEII, SOFA and NUTRIC scores, Glasgow coma scale and weight. In addition, the length of Intensive Care Unit stay, days of dependence on ventilator and 30-day mortality were studied. SPSS software (version 13.0) was used for data analysis.

**Results:**

Taurine significantly decreased the serum levels of IL-6 (*p* = 0.04) and marginally APACHEII score (*p* = 0.05). In addition, weight loss was significantly lower in taurine group (*p* = 0.03). Furthermore, taurine significantly increased the GCS (p = 0.03). The groups were not different significantly in terms of levels of IL-10, hs-CRP, and TNF-α, SOFA and NUTRIC scores, 30-day mortality, length of ICU stay and days of dependence on ventilator.

**Conclusion:**

According to the results of the present study, taurine supplementation can reduce the IL-6 levels as one of the important inflammatory markers in these patients; and enhances the clinical outcomes too.

**Trial registration:**

IRCT, IRCT20180514039657N1. Registered 22 June 2018.

## Background

Traumatic brain injury (TBI) is defined as the change in the brain function or other brain pathologic evidence caused by an external force [1]. It is one of the most common types of trauma and the main cause of death among various types of trauma [2–4]. Despite tangible developments in trauma management, the disease remains a great cause of mortality and is a serious problem for any society [5]. TBI occurs under two phases. Primary injury, which is the main determinant of outcomes, occurs at the time of the incident and is resistant to treatment. After that, physiological and pathophysiological reactions including the activation of inflammatory response involved in delayed cell death leading to a secondary injury and providing a chance for clinical interventions. The activation of inflammatory pathways in response to TBI leads to the release of pro- and anti-inflammatory cytokines and chemokines [6, 7]. Secondary injuries lead to inflammation in the nervous system and this process increases the nervous system damages [8]. TBI illustrates the pathophysiology of inflammation including brain edema and functional deficits in the nervous system [9]. If the inflammation is controlled for a specific period of time; it can be beneficial. While persistent or excessive inflammation is one of the main cause of nervous defects [10]. TBI is associated with increased levels of cytokines such as IL-6, IL-10, and TNF-α [11–13], which worsen the TBI condition and delay the improvement by generating oxidative stress and metalloproteinases [14, 15].

Taurine (2-aminoethane sulfonic acid) is a beta amino acid that is not involved in the structure of proteins and is found in the free form in the body. This is one of the most abundant free amino acids in the brain of mammals, which is essential to proper functioning of it [16, 17]. The synthesis of Taurine under stress can be reduced [18]. Taurine is influential in terms of detoxification, membrane stabilization, osmoregulation, nervous system, calcium homeostasis, inflammatory reactions, conjugation of bile acids, glucose metabolism, and anti-oxidant activity [16, 18–21].

Several studies have reported that taurine concentration decreases in response to trauma [22–25]. On the other hand, some studies have suggested the modification of inflammatory response by taurine supplementation [26–28].

To date, no investigation has focused on the efficacy of supplementation with taurine in TBI patients. Therefore, we carried out this study to assay the influence of taurine on this specific patient. The primary aims were to determine whether taurine affects the serum levels of IL-6, IL-10, TNF-α, and hs-CRP. Our secondary aim was specifying the effect of taurine on disease progression, length of ICU stay, duration of mechanical ventilation, 30-day mortality rate, weight, and nutritional risk.

## Material and methods

This double-blind randomized controlled trial (RCT) was performed on TBI patients, who were admitted to the ICU of Golestan Hospital in Ahvaz, the largest city in southwest of Iran. On the first day of ICU admission, the patients were assessed based on the inclusion and exclusion criteria and enrolled in the study if they were eligible. The study protocol was approved by the Ethics Committee of Ahvaz Jundishapour University of Medical Sciences. This study was also registered in the Iranian Registry of Clinical Trials (code: IRCT20180514039657N1). Because of the patients’ unconsciousness, informed consent was obtained from their families for inclusion in the study.

The patients (*n* = 22) were divided into two groups via randomization (ratio, 1:1). To assign the patients into each group, a randomized block method was applied with a block size of six patients. Randomization was carried out based on a computer-generated random sequence of numbers. The sealed envelopes containing the numbers were kept by an independent third party, who was not involved in the clinical conduct of this study until all data were collected and verified. The patients, as well as individuals who were engaged in recruiting the participants, assigning the interventions, and evaluating the results, were blind to the group assignments.

The inclusion criteria were TBI patients over the age of 18 years, receiving enteral nutrition, with a Glasgow Coma Scale (GCS) score of 6–12. The GCS score was determined based on the patient’s condition and the intensivist’s opinion. On the other hand, patients were excluded if they were pregnant or lactating; taking anti-inflammatory medications or corticosteroids before admission; or had liver disorders, kidney diseases, or congenital disorders affecting amino acid metabolism. Also, patients were excluded if more than 60% of their energy requirements were not provided by enteral nutrition in the first week.

The enteral nutrition was initiated on the first day of ICU admission. The control group received an Standard Entera Meal (Karen Pharma and Food Supplement Co., Iran), and the taurine group received 30 mg/kg/day of taurine powder (Nutricost, USA) at a maximum dose of 3 g/day for 14 days, in addition to the Standard Entera Meal. This dosage of taurine was selected based on previous studies on the safe and effective dose of taurine [29, 30]. Taurine powder was given to the patients in two divided doses. The third person, who was given the randomization sequence, prepared the patient’s gavage for each meal and delivered it to the nurse in a solution form. The appearance, taste, color, odor, and containers of the gavage solution were similar. The gavages were coded by the third person, and only he was aware of their contents.

The weight of the patients was measured upon entering the study, using Seca 984 bed and dialysis scale. The patients’ energy requirements were calculated to be 25 kcal/kg/day, based on the formula. The patient’s feeding started at 30 ml/h, and then, 30 ml/h was added every three hours to reach the calculated energy requirements within 48–72 h. For determining the nutritional risk, the Nutrition Risk in Critically Ill (NUTRIC) score was measured. The NUTRIC score is the first nutrition risk instrument that is specifically validated for ICU patients and can identify patients at risk of malnutrition [31].

The clinical history and demographic characteristics of the patients were determined upon admission to the ICU. To evaluate the disease progression (response to treatment), the Sequential Organ Failure Assessment (SOFA) and Acute Physiology and Chronic Health Evaluation II (APACHE II) scores, along with the GCS score, were computed for all patients on the 1st and 14th days of the study. Generally, the GCS score is the most commonly used clinical tool for evaluating the severity of neurological injury in adults [32] due to its acceptable inter-observer reliability and predictive validity [33]. Moreover, the APACHE II score is a tool to measure disease severity in patients hospitalized in ICU [34]. The SOFA score is an objective and simple score that allows measuring the number and severity of organ dysfunction; it is a suitable prognostic index within the first days of hospitalization in ICU [35].

To measure the serum levels of IL-6, IL-10, TNF-α, and hs-CRP on the 1st and 14th days, 10 mL of venous blood was taken from each patient, and after centrifugation and serum detachment, the serum was kept at − 70 °C until further analysis. The IL-6, IL-10, TNF-α, and hs-CRP levels were measured using the ELISA kits. Changes were calculated based on the difference in each variable on day 14 vs. day 1. The length of ICU stay, duration of mechanical ventilation, and 30-day mortality rates were also investigated.

### Statistical analysis

As specified by the preceding study on the serum levels of IL-6 variable [29], the total sample size of 30 patients was calculated by the averages comparison formula based on α = 0.01 and β = 0.1.

$$\mathrm{n}=\left\{{\left({\mathrm{Z}}_{1-\frac{\upalpha}{2}}+{\mathrm{Z}}_{1-\upbeta}\right)}^2\left({\mathrm{S}}_1^2+{\mathrm{S}}_2^2\right)\right\}/{\left({\upmu}_1-{\upmu}_2\right)}^2$$

Considering the probability of 30% dropout of patients during the study, the final sample size was considered 44 patients.

The data were analyzed using the statistical software program SPSS 13.0 (SPSS Inc., Chicago, IL, USA).

The Kolmogorov-Smirnov test was used to determine the distribution of quantitative data. Comparisons between mean of the groups were made by Independent Samples t-test if data distribution was normal. The comparisons of non-normally distributed data were performed using Mann-Whitney U test to compare the differences between the two groups. Comparisons of qualitative data were done by chi-squared test or Fisher’s exact test. Within-group comparisons were made using Paired-Samples t-test or its nonparametric equivalent (Wilcoxon). Outcomes were reported at 95% confidence intervals. The significance level was considered two-sided *p* < 0.05.

## Results

**Fig. 1:**
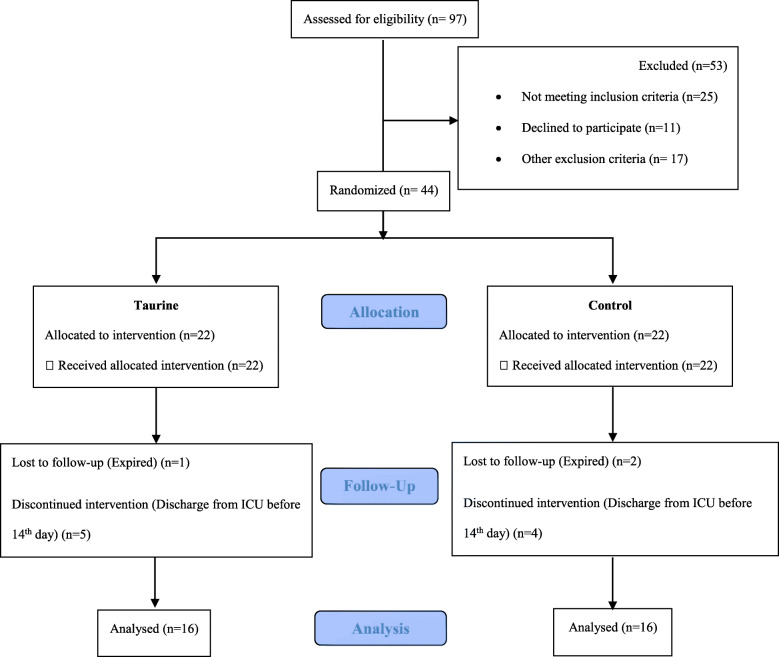
Flow diagram of the trial

From April to November 2018, 97 patients with TBI were assessed for eligibility and 44 were enrolled (Fig. 1); 32 patients completed the study, and others were excluded due to the reasons mentioned in Fig. 1.

Baseline characteristics of patients are shown in Table 1. No significant difference existed between the groups. It should be noted that according to the set criteria, all patients who entered the study had GCS = 6–9.

**Table 1:** Baseline characteristics of the study participants

Variable	Taurine (*n* = 22)	Control (*n* = 22)	*p-*value
Age (year)	33.3 ± 13.4	32.4 ± 11.4	0.80
Gender (N [%])			0.99
Male	17 (77.3)	18 (81.8)	
Female	5 (22.7)	4 (18.2)	
Weight (kg)	74.1 ± 11.72	74.3 ± 8.61	0.96
BMI (kg/ ***m***^**2**^)	25.3 ± 3.2	25.2 ± 2.5	0.85
Cause of TBI (N [%])			0.51
Accident	20 (90.9)	20 (90.9)	
Fall	1 (4.5)	2 (9.1)	
Gun shut	1 (4.5)	0 (0)	
Glasgow Coma Scale	7 (7, 7)	7 (7, 8)	0.07
Type of TBI based on CT scan findings (N [%])			0.79
Diffuse axonal injury	12 (54.5)	12 (54.5)	
Extradural hemorrhage	3 (13.6)	2 (9.1)	
Subarachnoid hemorrhage	2 (9.1)	3 (13.6)	
Subdural hematoma	4 (18.2)	4 (18.2)	
Intraventricular hemorrhage	0 (0)	1 (4.5)	
Intracerebral hemorrhage	1 (4.5)	0 (0)	
Surgical procedures			0.99
Craniotomy	10 (45.5)	10 (45.5)	
Temperature (°C)	37.3 ± 0.7	37.3 ± 0.9	0.93
Mean Arterial Pressure (mmHg)	92 ± 13.6	93.6 ± 11.8	0.68
APACHEII score	14 (12, 16)	14 (11, 16)	0.80
SOFA score	8 (6, 9)	8 (7, 9)	0.25

The Fisher’s exact test was used for gender, the Pearson chi-square test for cause of TBI, type of TBI and surgical procedures, Mann-Whitney U test for GCS, APACHEII and SOFA scores, and independent samples t-test for the others. Values are presented as mean ± SD, median (IQR), or frequency (percent).

Nutrition variables were assessed in each group and are presented in Table 2. Mean energy intake was compared with the energy requirement calculated by the formula. No significant difference existed between the groups for energy intake. Weight loss in the taurine group was significantly lower than the control group (*p* = 0.03). The changes of NUTRIC score showed no significant difference between the groups (*p* = 0.40), but it was decreased significantly in taurine group (*p* = 0.01).

**Table 2:** Nutritional variables of the study participants

Variable	Taurine (*n* = 16)	Control (*n =* 16)	*p*-value^*^
Energy intake (kcal/day)	1503.67 ± 111.04	1485.58 ± 136.28	**0.68**
The ratio of energy intake to the calculated energy requirement by formula (%)	85.55 ± 6.95	83.58 ± 10.09	**0.53**
Time to reach goal nutrition (day)	3 (3,4)	3 (3,4)	**0.88**
Weight			
Day 1	73.70 ± 13.06	75.96 ± 8.63	**0.57**
Day 14	68.9 ± 12.66	70.35 ± 8.24	**0.70**
Changes (1,14)	−4.80 ± 0.93	−5.61 ± 1.09	**0.03**
*p*-value^¥^	**0.0001**	**0.0001**	
NUTRIC score			
Day 1	2.5 (2,3)	2 (2,3)	**0.87**
Day 14	2 (1,2)	2 (1,2)	**0.36**
Changes (1,14)	−1(−2, −0.25)	−0.5(−1.75, 0.00)	**0.40**
*p*-value^¥^	**0.01**	**0.11**	

Values are presented as mean ± SD, median (IQR). Mann-Whitney U test was used for NUTRIC score and time to reach goal nutrition. Independent Samples T test for others. A group comparison was made using a paired-samples t-test for weight; this was made by Wilcoxon for the others. *p-value for comparison between groups; ^¥^ *p*-value for comparison within the group.

**Fig. 2:**
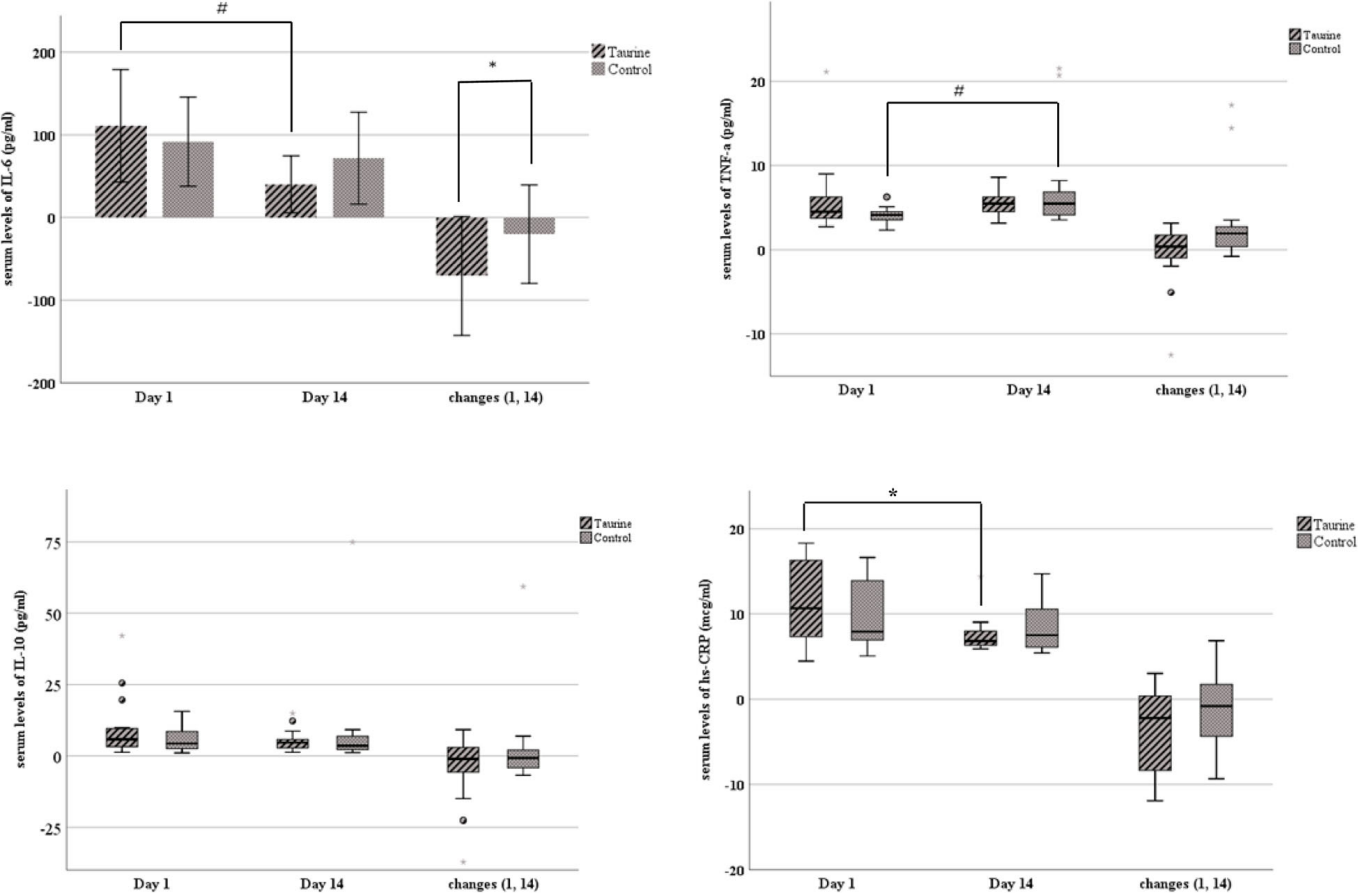
Serum levels of inflammatory markers. IL-6 shown as Mean ± SD, others as median (IQR). Independent Samples t-test was used for IL-6, and Mann-Whitney U test for others. A group comparison was made using a paired-samples t-test for IL-6; this was made by Wilcoxon for the others. ^*^ *P* < 0.05, ^#^ *P* < 0.01

Figure 2 shows the levels of inflammatory markers. At the beginning of the study, no significant difference excited between the groups for any of the inflammatory markers. The mean changes of serum IL-6 between the groups were significantly different (− 70.75 ± 71.89 vs. -20.09 ± 59.51, *p* = 0.04). There were no significant differences between the groups in levels of IL-10, hs-CRP, and TNF-α (*p* = 0.81, *p* = 0.24 and *p* = 0.07, respectively). However, the increase in TNF-α in the control group and the decrease in hs-CRP in the taurine group was significant after 14 days (*p* = 0.004 and *p* = 0.02, respectively).

Clinical outcomes are presented in Table 3. On the 1st day, no significant difference existed between the groups in GCS, APACHEll, and SOFA scores. The mean changes of GCS significantly increased by taurine supplementation (*p* = 0.03). The changes of APACHEll score showed marginal differences between two groups (*p* = 0.05). In other words, taurine decreased it. The SOFA score, mortality rate, duration on mechanical ventilation, and length of ICU stay were not different considerably between the groups (*p* = 0.06, *p* = 0.33, *p* = 0.26 and *p* = 0.96, respectively). However the SOFA score decreased significantly in Taurine group (*p* = 0.003).

**Table 3:** The clinical outcomes of study participants

Variable	Taurine (*n* = 16)	Control (*n =* 16)	*p*-value^*^
APACHEII score			
Day 1	14 (12, 17)	13 (11, 15)	**0.34**
Day 14	12 (9, 13)	13 (9, 14)	**0.31**
Changes (1, 14)	−4 (−6, −1)	−1 (− 4, 2)	**0.05**
*p-*value^¥^	**0.003**	**0.32**	
SOFA score			
Day 1	8 (6, 9)	8 (6, 9)	**0.56**
Day 14	5 (4, 6)	7 (5, 8)	**0.02**
Changes (1, 14)	−3 (−4, −1)	−1 (−3, 1)	**0.06**
*p-*value^¥^	**0.003**	**0.06**	
GCS			
Day 1	7 (7, 7)	7 (7, 8)	**0.18**
Day 14	9 (7, 10)	8 (6, 9)	**0.17**
Changes (1, 14)	2 (0, 3)	0.0 (−1, 1)	**0.03**
*p-*value^¥^	**0.002**	**0.30**	
30-day Mortality (N (%))			**0.33**
Yes	1 (6.3)	4 (25)	
No	15 (93.8)	12 (75)	
Days on mechanical ventilation	18 ± 9	23 ± 14	**0.26**
Length of ICU stay	22 (17, 31)	21 (18, 39)	**0.96**

Values are presented as mean ± SD, median (IQR) or Frequency (percent). Independent Samples T test was used for days on mechanical ventilation, Fisher’s exact test for 30-day mortality and Mann-Whitney U test for others. Within group comparison was made by by Wilcoxon for APACHEII and SOFA scores and GCS. *p-value for comparison between group; ^¥^ *p*-value for comparison within group.

## Discussion

The present study evaluated the effects of taurine on the clinical outcomes of patients with sustained TBI. The results revealed the beneficial effect of taurine supplementation on the serum concentration of IL-6, APACHE II score, and GCS score.

One of the most important ways to increase immunity and reduce the likelihood of adverse outcomes in patients admitted to the ICU is to use enteral immunonutrition [36]. Over the years, researchers have studied the effects of various nutrients, such as omega-3 and glutamine, which can boost the immune system in critically ill patients. Taurine is a sulfur-containing amino acid, which has been evaluated as an immunomodulator [37, 38]. However, to the best of our knowledge, it has not been evaluated in patients with TBI. Previous studies indicated that the plasma taurine levels declined in critically ill patients. In this regard, Vermeulen et al., in an observational study, found that in patients admitted to the ICU, the plasma concentration of taurine decreased by more than 50% in the first 5 days of admission, which was associated with a high lactate level and a longer duration of mechanical ventilation and ICU stay [25].

Evidence suggests that the overexpression of inflammatory cytokines, such as IL-6, TNF-α, and hs-CRP, plays an important role in the pathogenesis of TBI [39]. In these patients, the acute inflammatory response varies in the early and late stages [40]. Also, within a short time after brain injury, abundant production of pro-inflammatory cytokines, such as IL-6 and CRP, occurs [41]. The role of inflammatory cytokines, especially TNF-α, IL-1β, IL-6, and IL-8, is well-established in neurodegeneration and cognitive deficits. Recently, Su et al. reported that the level of hs-CRP was associated with cognitive impairments following TBI [42].

The addition of taurine to a nutritional formula may reduce neuroinflammation and improve the clinical and nutritional status of TBI patients. The use of inexpensive, safe, and natural supplements is considered as an adjuvant therapy to inflammation control in these patients. As mentioned earlier, taurine is a beta-amino acid with anti-inflammatory and antioxidant activities in the central nervous system [43, 44]. However, no clinical trial has been conducted on taurine in patients with TBI so far. Our findings demonstrated that supplementation with taurine caused a significant decrease in the serum levels of IL-6 and hs-CRP and also prevented a further increase in the level of TNF-α; nevertheless, it exerted no effects on the IL-10 level.

In line with our findings, in a RCT, the administration of taurine, in addition to enteral nutrition, resulted in a decrease in the levels of IL-6 and CRP in critically ill septic patients [29]. Also, in a study on rats with TBI, 200 mg/kg/day of taurine for 1 week reduced the levels of IL-6, IL-10, and TNF-α [45]. Similar findings have been also reported in some animal studies [46–49]. However, contrary to our findings, a previous study on burn patients showed that the enteral administration of taurine significantly reduced the IL-10 levels, but did not affect the serum levels of hs-CRP and TNF-α [50]. Besides, in another study on elderly people with hip fractures, supplementation with 6 g/day of taurine had no effects on inflammatory markers, length of hospital stay, and mortality [51]. One of the reasons for the discrepancy between the results of different studies is the difference in the study population and the type of disease.

The mechanisms through which taurine acts as an anti-inflammatory agent have not been clearly defined. It is known that activated inflammatory cascades damage the blood-brain barrier (BBB) after TBI, which leads to the entry of circulating immune cells to the injured site and directly affects neuronal survival and death [52–56]. It has been shown that these activated cells release mediators, such as free radicals and pro-inflammatory cytokines [14]. Also, some studies have suggested the ability of taurine to suppress the NF-κB signaling pathway. It seems that taurine supplementation can exert inhibitory effects on NF-κB by oxidation of IĸB-α as an inhibitory protein [57].

Moreover, the anti-inflammatory activity of taurine may be attributed to its antioxidant ability to neutralize hypochlorous acids, which are reactive molecules in mammalian neutrophils, as well as monocytes, produced through the myeloperoxidase pathway [58], by forming taurine chloramine, as a relatively more stable and less toxic compound. Taurine chloramine can be generated at the inflammation site and control the expression and release of cytokines, such as nitric oxide, IL-6, IL-8, and TNF-α [59]. Since cytokines are mostly secreted by reactive astrocytes, inhibition of astrocytes may be another mechanism that regulates the cytokine levels [45].

Our findings related to the clinical outcomes of the patients showed that taurine supplementation decreased weight loss, APACHE II score, and SOFA score, while increasing the GCS score; however, it had no effects on the length of ICU stay, duration of mechanical ventilation, and 30-day mortality. Since APACHE II and SOFA scores represent the performance of different organs, a reduction in these scores (even if classifications for mortality prediction do not change) can indicate progress in recovery through reduction and control of organ dysfunction. Similar to our findings, an experimental model of head injury showed that taurine supplementation at 15 and 50 mg/kg/day decreased weight loss [60]. Since limited studies have been carried out in this area, the mechanism of action is not clear, and further studies are needed in the future to investigate the mechanisms of its effect. Nevertheless, the observed effect may be related to the reduction of inflammation and oxidative stress in these patients, leading to reduced weight loss in the intervention group.

According to our literature review, there was no study evaluating the effects of taurine supplementation on the GCS, APACHE II, and SOFA scores. Although differences in the length of ICU stay, duration of mechanical ventilation, and 30-day mortality were not statistically significant, they might be clinically important and reduce the imposed costs on the healthcare system. Also, changes in these clinical data are usually statistically significant in studies with a large sample size. Therefore, future studies with a larger sample size are needed to determine the effects of taurine on the clinical improvement of patients with TBI.

The present study had several strengths, which made the results more reliable. First, this study was the first trial evaluating the effects of taurine supplementation on patients with TBI. Second, the dosage of taurine used in this study was well-tolerated by the patients, and no side effects were reported.

On the other hand, the limitations of this study were its small sample size, short duration of supplementation, lack of measurement of the plasma taurine concentration due to limited finances, and lack of resting energy expenditure (REE) measurements due to the absence of necessary equipment. Since this study is the first clinical trial evaluating the effects of taurine supplementation on TBI patients, there was no similar study in the literature to review and compare the clinical outcomes. Therefore, it is necessary to conduct further studies with a larger sample size and longer supplementation periods while measuring the plasma concentration of taurine to confirm the use of this agent for TBI patients to improve inflammation and clinical outcomes.

## Conclusion

According to the findings of this study, taurine supplementation in TBI patients was followed by clinical benefits, because it is reduced the levels of IL-6, APACHEII score, and weight loss.

Furthermore, GCS of patients in the taurine group improved significantly. Therefore, taurine can reduce the inflammation of patients with TBI and may be considered as an adjunctive treatment for these patients. However, more studies with larger sample size are needed to highlight the taurine advantages for this group of patients.

## Data Availability

The materials of this study are available at https://en.irct.ir/trial/31173.

## Abbreviations

TBI: Traumatic brain injury
ICU: Intensive Care Unit
BBB: Blood Brain Barrier
GCS: Glasgow coma scale
SOFA: Sequential Organ Failure Assessment
APACHEII: Acute Physiology and Chronic Health Evaluation II
NUTRIC: Nutrition risk in critically ill
IL-6: Interleukin 6
IL-10: Interleukin 10
hs-CRP: High sensitive C-Reactive Protein
TNF-α: Tumor Necrosis Factor-alpha
IQR: Interquartile range
SD: Standard deviation
NF-κB: Nuclear factor kappa B
IĸB-α: Inhibitory kappa B alpha
